# Large-scale collection and annotation of gene models for date palm (*Phoenix dactylifera*, L.)

**DOI:** 10.1007/s11103-012-9924-z

**Published:** 2012-06-27

**Authors:** Guangyu Zhang, Linlin Pan, Yuxin Yin, Wanfei Liu, Dawei Huang, Tongwu Zhang, Lei Wang, Chengqi Xin, Qiang Lin, Gaoyuan Sun, Mohammed M. Ba Abdullah, Xiaowei Zhang, Songnian Hu, Ibrahim S. Al-Mssallem, Jun Yu

**Affiliations:** 1 Joint Center for Genomics Research (JCGR), King Abdulaziz City for Science and Technology (KACST) and Chinese Academy of Sciences (CAS), Riyadh, Kingdom of Saudi Arabia; 2 CAS Key Laboratory of Genome Sciences and Information, Beijing Institute of Genomics, Chinese Academy of Sciences, Chaoyang District, Beijing, China; 3 Graduate University of Chinese Academy of Sciences, Shijingshan District, Beijing, China; 4 Department of Biotechnology, College of Agriculture and Food Sciences, King Faisal University, Al-Hssa, Hofuf, Kingdom of Saudi Arabia

**Keywords:** *Phoenix dactylifera*, cDNA, Pyrosequencing, Gene models

## Abstract

The date palm (*Phoenix dactylifera L.*), famed for its sugar-rich fruits (dates) and cultivated by humans since 4,000 B.C., is an economically important crop in the Middle East, Northern Africa, and increasingly other places where climates are suitable. Despite a long history of human cultivation, the understanding of *P. dactylifera* genetics and molecular biology are rather limited, hindered by lack of basic data in high quality from genomics and transcriptomics. Here we report a large-scale effort in generating gene models (assembled expressed sequence tags or ESTs and mapped to a genome assembly) for *P. dactylifera*, using the long-read pyrosequencing platform (Roche/454 GS FLX Titanium) in high coverage. We built fourteen cDNA libraries from different *P. dactylifera* tissues (cultivar Khalas) and acquired 15,778,993 raw sequencing reads—about one million sequencing reads per library—and the pooled sequences were assembled into 67,651 non-redundant contigs and 301,978 singletons. We annotated 52,725 contigs based on the plant databases and 45 contigs based on functional domains referencing to the Pfam database. From the annotated contigs, we assigned GO (Gene Ontology) terms to 36,086 contigs and KEGG pathways to 7,032 contigs. Our comparative analysis showed that 70.6 % (47,930), 69.4 % (47,089), 68.4 % (46,441), and 69.3 % (47,048) of the *P. dactylifera* gene models are shared with rice, sorghum, *Arabidopsis,* and grapevine, respectively. We also assigned our gene models into house-keeping and tissue-specific genes based on their tissue specificity.

**Electronic supplementary material:**

The online version of this article (doi:10.1007/s11103-012-9924-z) contains supplementary material, which is available to authorized users.

## Background

Date palm, *Phoenix dactylifera* L., gains a considerable fame for its fruits (dates), which have an excellent capability to enrich sugar in mesocarp up to 50 % of the total weight (Bourgis et al. 2011). *P. dactylifera* used to play important roles in human civilization: its fruits were taken as staple food, its trunks were used as architectural materials, and palm trees themselves were and still are used as ornamental plants. *P. dactylifera* grows mostly in arid climates and deserts. It may evolve to have a unique set of genes and regulatory mechanisms to cope with climates and develop resistance to abiotic and biotic stresses.

The advancement of the next-generation (next-gen) sequencing technologies allows us to generate cDNA or ESTs (expressed sequence tags) in large-scale and a cost-effective way. Among the three major next-gen sequencing platforms, the Roche/454 pyrosequencing platform (GS FLX Titanium) produces long reads (~400 bp) that are readily assembled into large contigs albeit limited in numbers due to higher cost than other platforms. Illumina HiSeq2000 and Life Technologies SOLiD systems are able to generate huge numbers of reads per machine run but shorter read lengths. Both platforms, however, are powerful for detecting low-abundant transcripts and validating homoploymeric nucleotide tracts that are less abundant but remain ambiguous in the Roche/454 data. Therefore, a combined dataset, one part from the longer reads and the other part from coverage, is of importance for better characterization of transcriptomes; examples are numerous, such as datasets from Medicago (Benedito et al. 2008), olive (Alagna et al. 2009), mangroves (Dassanayake et al. 2009), and chickpea (Hiremath et al. 2011), as well as those from animals and humans (Hubbard et al. 2005; He et al. 2008).

Armed with the next-gen sequencing tools, we are in a unique position to significantly improve our understanding of *P. dactylifera* genomics and biology. There have been only two significant reports on *P. dactylifera* genomic data based on the next-gen sequencing technologies; one concerns a draft genome sequence assembly (Al-Dous et al. 2011) and the other is a comparative analysis between the fruits of oil palm and *P. dactylifera*, where mechanisms of carbon partition were explored in depth (Bourgis et al. 2011). Here we report an in-depth transcriptomic sequencing effort to build *P. dactylifera* gene models based on data from different tissues and at several developmental stages. We generated 30,854 of annotated gene models that are treated as putative full-length cDNAs and compared them to the latest data from other plant species, including rice (49,066; RGAP7, October 31, 2011) and *Arabidopsis thaliana* (37,761; TAIR9); the amount of gene models is comparable for an initial annotation of plant genomes.

## Results and discussions

### Sequence acquisition and assembly

We used RNA samples from eight tissues (young leaf, mature leaf, root, fruit, flowers and offshoots from both male and female trees) for a thorough gene discovery effort. Since we already made a series of cDNA libraries from the developing fruits and sequenced them previously (seven fruiting-stage-specific cDNA libraries; Yin et al. 2012), we made seven additional cDNA libraries from the seven non-fruit tissues. We generated 7,955,347 raw sequence reads from the seven libraries, which are equal to over one million reads per library. Adding these raw reads from the seven fruit-specific libraries and 7,823,646 reads from them, we now have data from 14 libraries that contain 15,778,993 raw sequence reads (Table 1). All sequence data were deposited in SRA (Sequence Read Archive) of GenBank and the accession numbers for the two groups of datasets are SRA049307 and SRX096040. The quality of the data is rather satisfactory, where more than 92 % of the raw reads are in a range of 300–650 bp in length (Supplementary Fig. S1). Even after the removal of low-quality data (including reads shorter than 100 bp and adaptor tag sequences), we still have 14,435,855 (91.4 %) reads for subsequent sequence assembly procedures.

**Table 1:** Summary of sequence assembling, data processing and annotation

Raw data	Number
Raw reads	15,778,993
Average read length (bp)	352
Assembled reads	14,435,855
Average read length (bp)	345
*Assembling results*	
Before processing/after processing	
Contigs	83,240/67,651
Total contig length (bp)	131,955,922/102,128,874
Average contig length (bp)	1,585/1,510
The largest contig length (bp)	16,000/16,000
Contig N50 (bp)	2,042/1,911
GC content of contigs	44,17 %/44.27 %
Singletons	755,503/301,978
Average singleton length (bp)	213/348
Total singleton length (bp)	161,174,728/104,941,945
Annotation	
Contigs annotated based on the plant UniProt database	52,090
Contigs annotated based on the NR database	635
Gene models	30,854
Contigs annotated with GO terms	36,086
Contigs annotated with KO identifiers	7,032
Contigs assigned with EC numbers	5,727

We designed a specific pipeline for contig assembly (Supplementary Fig. S2A), which has two components: read assembly and quality filters. In the first step, we pooled all reads together and assembled them using the Roche/454 de novo assembler (Version 2.6) after the removal of mitochondrial and chloroplast sequences (Yang et al. 2010). This step yielded 83,240 contigs and 755,503 singletons with an average size of 1,585 and 213 bp, respectively (Table 1). Although the number of singletons appear quite larger but they are still a small fraction of the total reads, merely 5 %.

In the second step, we processed contigs and singletons. First, we eliminated sequences that are shorter than 150 bp, which are composed of 545 contigs and 333,128 singletons. Second, we used BLASTn search against the microbial databases, and the search identified 1,650 contigs and 18,359 singletons, which are either viral or bacterial origins. Third, we identified 13,178 contigs as redundant sequences because they are covered by other larger ones with high identity (95 %) and significant coverage (90 %), based on Blast results. Fourth, we further annotated ribosomal RNA sequences based on BLASTx search against a ribosomal RNA database (Pruesse et al. 2007), and 216 contigs and 5,805 singletons have significant hits. Fifth, all non-redundant and contamination-free singletons are compared with 67,651 contigs using BLASTn. This revealed that 96,234 singletons were also completely covered by our contig collections. Finally, we still have 301,978 singletons left for further annotations. Nevertheless, we took the high-coverage contigs as gene models, which have a length range of 150 to 16,000 bp. The average and N50 lengths of the gene models are 1,510 and 1,911 bp, respectively (Supplementary Fig. S3).

### Analysis of the non-redundant sequences and gene models

**Fig. 1:**
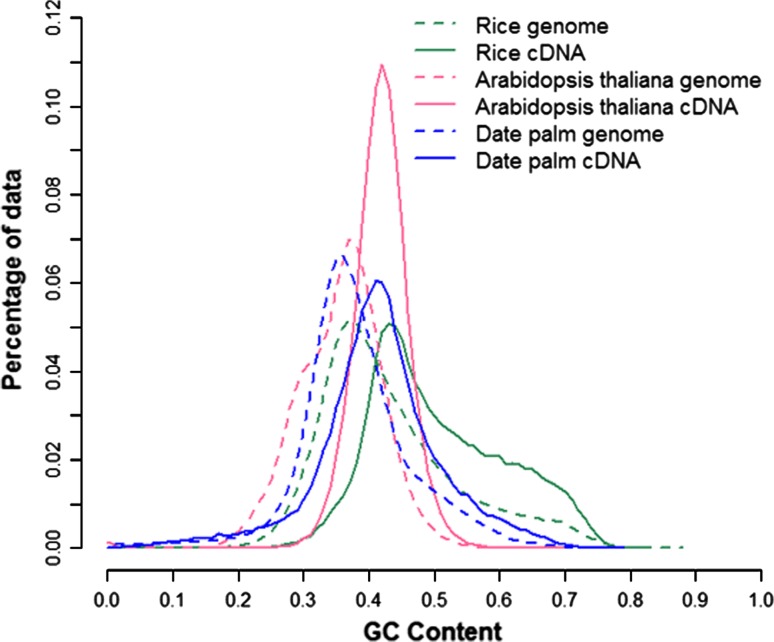
The distribution of GC contents in genomes and CDS (coding sequences) of date palm, rice, and Arabidopsis. The genomic GC content was calculated in a 500-bp window in a step size of 500 bp over concatenated sequences. The GC content of cDNAs was calculated in a similar way

We predicted ORFs for all gene models using “getorf” (EMBOSS 6.2.0). A total of 67,648 (99.9 %) putative gene models were predicted to have ORFs, and of the total, we have 49,284 (72.8 %) gene models with ORFs longer than 100aa (amino acids). Comparing the predicted ORFs for all gene models to protein-coding data collected from the Plant UniProt database, we identified 30,854 and 3,047 gene models to have complete and partial CDSs, respectively. We regard the full-length CDSs as full-length gene models since we also validated them *in sillico* by mapping to the *P. dactylifera* genome assembly (data not shown). The gene models with complete CDSs showed average GC contents for 5′ untranslated regions (5′-UTRs), ORFs, and 3′-UTR as 43.4, 48.7, and 42.3 %, respectively (Fig. 1). We calculated the GC content using a 500-bp window, because this smaller window is more informative. When the window size is larger than a typical gene size, differences between intergenic sequences and genes became obscure (oryza et al. 2002). We also calculated the codon usage based on the gene models in comparison with those of rice and *Arabidopsis* (Supplementary Table S1).

We also analyzed the repeat contents of the full-length gene models and found 2,043 simple sequence repeats (SSR) (repeat length ≥20 bp) using a perl script MISA (MIcroSAtellite identification tool; http://pgrc.ipk-gatersleben.de/misa/). The most abundant SSRs are di-nucleotides (47.8 %), followed by tri- (42.1 %), tetra- (6.5 %), penta- (1.8 %), and hexa- (1.5 %) nucleotide repeats (Supplementary Table S2). We also noticed a strong bias between coding regions and UTRs; 55.5 % of the tri-nucleotide repeats occurred in coding regions and 87–98.6 % of di-, tetra-, and penta-nucleotide repeats were found in 5′ and 3′ UTRs. Among the non-redundant 67,651 gene models, we detected a total of 4,178 known transposable elements (TEs) using tBLASTx search (*E* value ≤ 10^−10^) against a transposable elements database (http://www.girinst.org), and most of the TEs (62.6 %) are DNA transposons and the rest are either LTR (long terminal repeat)-retrotransposons (32 %) or non-LTR-retrotransposons (5 %; Supplementary Table S3).

### Functional annotation of the *P. dactylifera* gene models

**Fig. 2:**
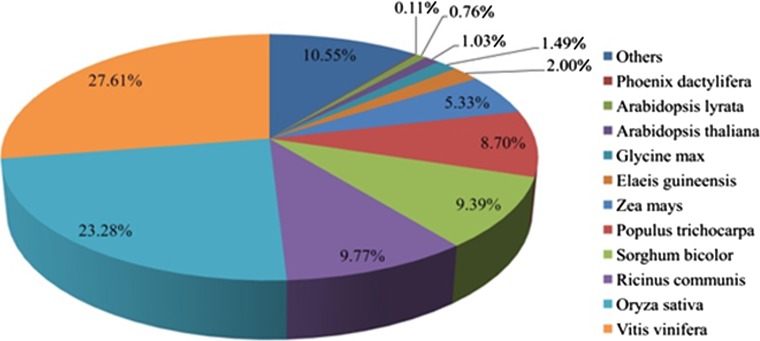
A species-based distribution of blast hits. The results are sorted according to the hits in an increasing way

We custom-designed an annotation pipeline for our data (Supplementary Fig. S2B). First, we aligned all gene models against the plant UniProt and NR database using BLASTx (*E* value ≤ 1 × 10^−5^) and 52,725 of the *P. dactylifera* gene models (77.93 % of the total gene models) have matches. The fraction of annotated sequences is higher than those (20 % to 40 %) previously reported for other de novo eukaryotic transcriptomes (Vera et al. 2008; Meyer et al. 2009; Haegeman et al. 2011). We provide two reasonable explanations for the higher annotation rates. First, the large amount of raw data we acquired allowed us to assemble longer cDNA sequences than the previous studies. Second, more plant genome sequencing projects have been completed so that we can take advantage of much more and better references. The best-hit species distribution of Blast matches is shown in Fig. 2. Approximately 27.6 % of the sequences have significant matches with grape (*Vitis vinifera*) sequences, followed by those of rice (*Oryza sativa*) and ricinus (*Ricinus communis*). The latter two species contributed to 23.28 and 9.77 % of the gene model annotations, respectively.

We searched all unannotated gene models against the Pfam database (Finn et al. 2010) and found 45 gene models containing functional domains. Next, we further annotated the gene models based on Gene Ontology (GO) and Kyoto Encyclopedia of Genes and Genomes (KEGG) databases (Moriya et al. 2007), and found that 36,086 (69 %) of the gene models can be assigned with GO terms and that 7,032 of them can be assigned with KEGG numbers (Supplementary Table S1). The distribution of the ten most abundant GO terms for the three GO root nodes—biological process, molecular functions, and cellular components—are listed in Table S4. In biological process, “primary metabolic process” and “cellular metabolic process” are the two largest subcategories, accounted for 41.2 and 39.9 % of the total, respectively. In molecular function, “nucleic acid binding” (22.4 %) and “nucleotide binding” (21.7 %) are the two most abundant subcategories, whereas “cell part” (39.6 %) and “intracellular” (27.8 %) are the most abundant subcategories in cellular component.

**Fig. 3:**
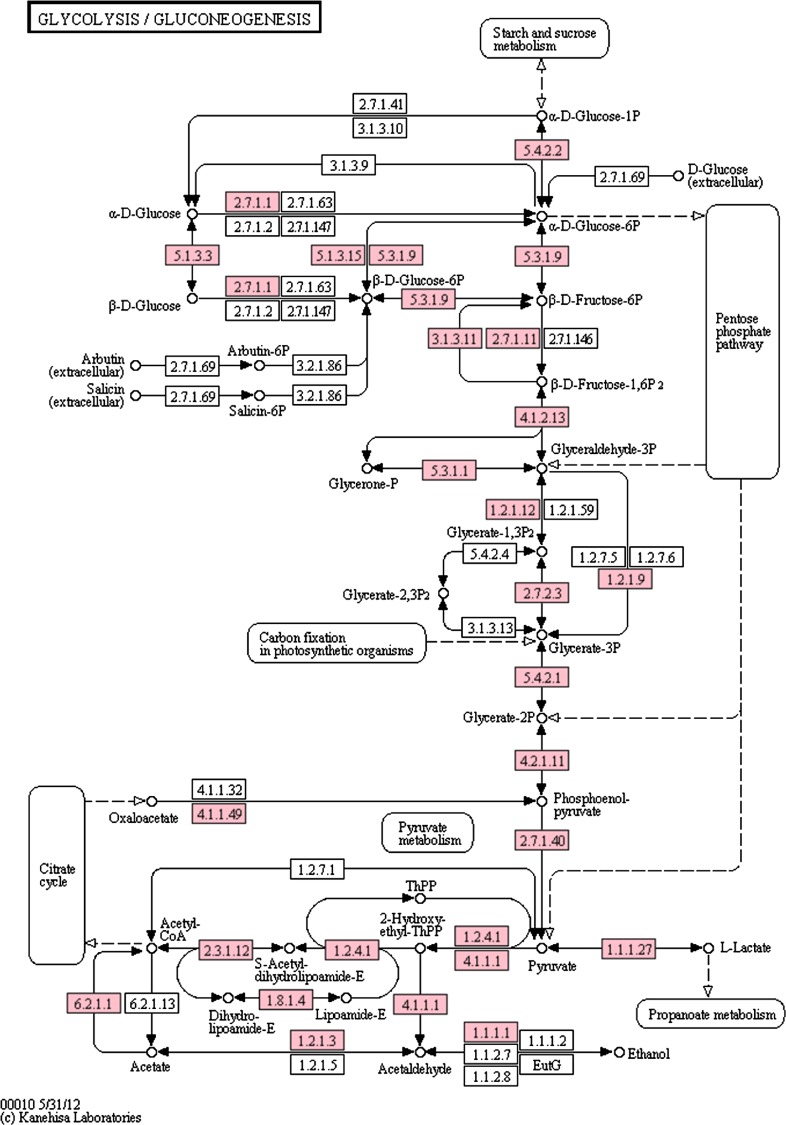
The distribution of *P. dactylifera* gene models (*pink*) in the glycolysis/gluconeogenesis pathway based on Arabidopsis genes

**Fig. 4:**
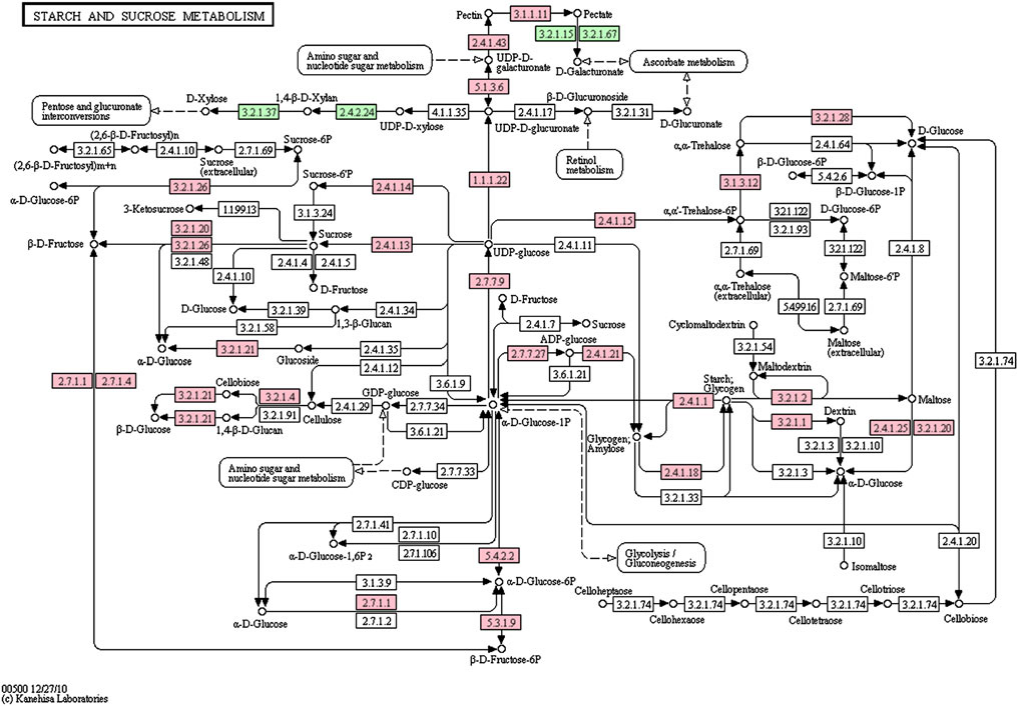
The distribution of *P. dactylifera* gene models in starch and sucrose metabolism pathways of Arabidopsis. The genes involved in the pathways are color-coded: *pink*, genes identified in our data; and *green*, genes involved in the pathway present in Arabidopsis but undetectable in our data

We also annotated 7,032 gene models with KO number to BRITE functional hierarchies, and 5,727 of them were assigned with EC number (Supplementary Table S1). The BRITE functional mapping revealed the most common classifications and categorized the gene models into 279 KEGG pathways, including pathways with essential functions for plant development, such as glycolysis/gluconeogenesis, citrate cycle (TCA cycle), photosynthesis, starch and sucrose metabolism, and cell cycle. Since our samples used in this study include a broad collection of tissues at multiple developmental stages, we were able to have an overview on gene expression profiles for basic metabolic processes during *P. dactylifera* development. There are 3,721 (52.9 %) gene models categorized into “metabolism”, as well as its subcategories, including “carbohydrate metabolism”, “amino acid metabolism”, “energy metabolism”, “lipid metabolism”, “nucleotide metabolism” and “glycan biosynthesis and metabolism”. In details, we further investigated the mapping result of our gene models against the “glycolysis/gluconeogenesis” pathway (using the Arabidopsis genome map as a reference) as an example (Fig. 3). Our data confirmed every single gene that participates in this pathway. The glycolysis/gluconeogenesis pathway has been recently identified as the most activated biochemical pathway during cassava storage root development, and plays a key role in starch accumulation (Yang et al. 2011). In another important pathway “starch and sucrose metabolism” (Fig. 4), our gene models cover almost all related genes with only three exceptions. In addition, all the key enzymes for starch and sucrose metabolism were detected, such as sucrose synthase (E2.4.1.13) (Winter and Huber 2000; Coleman et al. 2006), sucrose-phosphate synthase (Winter and Huber 2000), α-amylase (E3.2.1.1), β-amylase(E3.2.1.2) and starch synthase (E2.4.1.21) (Tetlow et al. 2004; Smith et al. 2005). Therefore, our gene models provide an excellent presentation of genes related to basic metabolic processes during date palm development. Other than “metabolism”, 2,318, 511, and 997 gene models fall into the categories including “genetic information processing”, “environmental information processing”, and “cellular processes” categories, respectively. The coverage of pathways by the annotated genes is satisfactory with only sporadic exceptions.

Our effort for the identification of transcription factors (TFs) resulted in 1,368 putative TF genes from 67,651 gene models. Although there are more TFs found in other plant species than *P. dactylifera*, 2,438 in rice and 2,023 in Arabidopsis, our result is acceptable for three reasons. The first reason is that we annotated our gene models based on homology to other data and certain fraction of the genes may not have been identified (such as with identities lower than the threshold). The second reason may be the incompleteness of our collection since both rice and Arabidopsis are well studied and for much longer periods. The third reason is the limitation of sampling depth with the particular sequencing platform we used here. We are of course in the process to generate much deeper coverage of the transcriptomes for the following accurate quantitative analysis.

Among the *P. dactylifera* TFs, we identified the bHLH (9.36 %), MYB (6.21 %), C2H2 (5.99 %) families as more abundant. The top ten *P. dactylifera* TF families are similar to those of rice and Arabidopsis (Supplementary Table S5). We noticed that the C3H family has slightly higher percentage in *P. dactylifera* (5.26 %) as compared to both Arabidopsis (2.77 %) and japonica rice (3.36 %), whereas the representation of the NAC family is relatively lower (4.75 %) than in Arabidopsis (6.67 %) and japonica rice (7.63 %). Among the less frequently found TF families, the CO-like, Trihelix, ARR-B, and Dof families showed relatively higher abundance in *P. dactylifera* when compared to the other two well-studied plants. Moreover, we further investigated tissue-specificity of the expressed TF families (Supplementary Table S6) and noticed that there is an obvious representation bias between male and female flowers. A total of 91 specifically expressed TF genes in male flower appear clustered into 13 TF families with very high coverage. Of the 91 TFs, the WRKY group (21.9 %) is most abundant, followed by the FAR1 (10.9 %) and ERF (10.9 %) families. Meanwhile, there are only 18 TF genes found in female flowers, which are clustered into 13 TF families with very low coverage (Supplementary Table S6). According to the new data on the WRKY family, often acting as repressors as well as activators, members of this gene family play significant roles in both repression and de-repression of important plant processes (Rushton et al. 2010). We assume that the specifically high-level expression of WRKY genes in male flower may also repress or activate certain biological processes in *P. dactylifera*.

The distribution of TFs in fruits is different. There are 267, 107, and 116 TFs found at different fruiting stages, fruit-I, fruit-II, and fruit-III, respectively, which are clustered into 38, 35, and 32 TFs families. Only 27 TF families are shared among the three developmental stages. Although the amount of TF families present in the three developmental stages are not large, more highly-expressed TF genes were found in the stage of fruit-I. In addition, some TF genes were only found in fruit-I and fruit-II; for example, HD-ZIP family genes are highly expressed in fruit-I and fruit-II, but not in fruit-III. And HD-ZIP is one of the TF family unique to plants and is classified into four subfamilies, according to a set of distinctive features (Ariel et al. 2007). The exact functions of these HD-ZIP TFs remain to be revealed in *P. dactylifera*.

Because the sequencing depth for male and female offshoots is different, the distribution of the identified TFs in the two offshoots appeared differently at two levels. First, among 210 TF genes identified in the female offshoot, only 42 of them were found in the male counterpart. These 210 TF genes are cluster into 38 TF families and the most abundant groups are WRKY (10.4 %) and ERF (10.4 %), followed by FAR1 (8 %) and C3H (6 %). Second, the distribution of TF families in female offshoot is similar to what in male flower, except the percentage of the WRKY group in female offshoot is less than that in male flower. Although offshoot planting is a traditional method for data palm propagation and the offshoots developed from the trunk of the mother plant produced fruits with the same quality as the mother palm (FAO), seed-based propagation is still viable if plant sex is identified at an early stage. Our dataset, coupled with high-coverage expression studies, is valuable for future research to understand *P. dactylifera* flower development and sex determination.

### Homology analysis

**Fig. 5:**
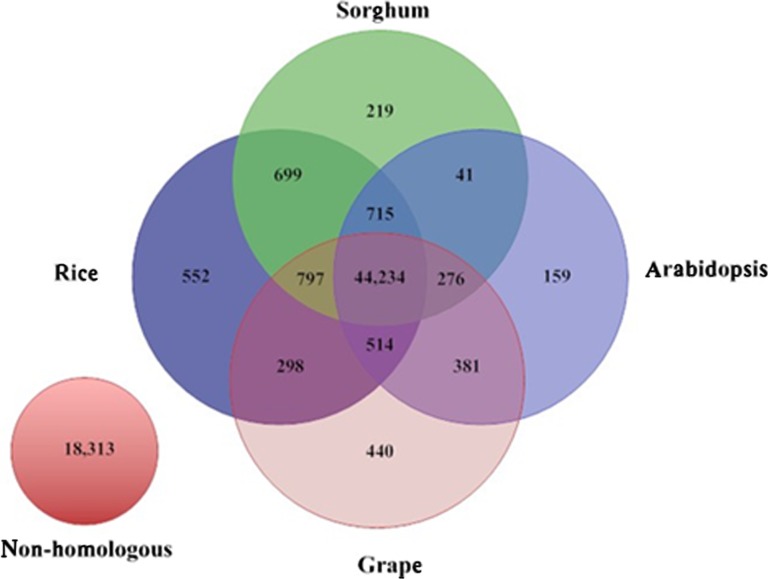
Homology analysis of *P. dactylifera* gene models in comparison with those of rice, sorghum, Arabidopsis, and grapevine. The 49,554 date palm gene models were aligned against annotated genes or gene models of each species based on BlastX, and the overlapping matches between species were displayed in Venn diagram. Two overlaps (108 overlapping hits between grape and sorghum, 121 overlapping hits between rice and Arabidopsis) are not represented in the diagram

Base on the best-hit definition (the best-hits sequence is orthologous), we compared the *P. dactylifera* sequences with the annotated genes from two monocot plants, *O. sativa* (japonica) (Hsing 2005; Ouyang et al. 2007) and *S. bicolor* (Paterson et al. 2009), and two dicot plants, *A. thaliana* (Arabidopsis and Initiative 2000) and *V. vinifera* (Jaillon et al. 2007). Using BLASTx (*E* value ≤ 1 × 0^−10^), we matched 70.6 % (47,930) and 69.4 % (47,089) *P. dactylifera* gene models to their orthologs in the monocots, rice and sorghum, respectively, as well as 68.4 % (46,441) and 69.3 % (47,048) to the dicots. A total of 49,554 gene models (73.0 %) showed orthology with at least one in all four datasets, 44,234 (65.2 %) in all four datasets, and 18,313 (27.0 %) with no homology to the annotated genes (Fig. 5). We further evaluated the no-match genes by mapping them to our *P. dactylifera* genome assembly (unpublished data) and found that almost all gene models (62,549) were aligned to the genome, using BLAT (identity ≥95 % and coverage ≥90 %) (Kent 2002). Among the gene models matched to the reference genome, 14,880 do not match to any other selected plant genes, and these gene models may represent unique genes to date palm, given the concern that homology-based analysis is always limited by parameters chosen for the analysis.

We also downloaded three publicly available related datasets for our comparative analysis. The first one is the *P. dactylifera* genes (the latest version from Weill Cornell Medical Collage in Qatar; http://qatar-weill.cornell.edu/research/datepalmGenome/index.html) that were predicted based on gene prediction software from genome sequences. The second one is cDNA contigs from oil palm (*Elaeis guineensis*) fruits and leaves, and the third cDNA contigs are from *P. dactylifera* fruits. The last two datasets were produced by an international team from France, the US, Tunisia, and Cameroon (Bourgis et al. 2011). We compared our *P. dactylifera* gene models to these datasets using BLASTn (*E* value ≤ 1 × 10^−10^; Supplementary Table S12), and 49,622, 50,834, 48,329, and 26,440 gene models matched with the predicted *P. dactylifera* genes from the Qatar team, as well as the *P. dactylifera* fruit, *E. guineensis* fruit and leaf cDNAs from the French-led team, respectively. These datasets are of importance for us to confirm our gene models although the matching rates are variable depending upon their sampling coverage. Furthermore, our data appear to have more genes than the other datasets. We not only have samples taken from various tissues and developmental stages but also collected a much larger amount of raw reads for the assembly*.* Compared with the predicted genes by the Qatar team, we have 18,029 more gene models, and 11,379 (63.1 %) of them are not yet annotated based on the known plant gene sets, which may represent novel or diversified genes in *P. dactylifera.* We summarized the detailed results from our comparative analysis at gene level in Table S13.

### Identification of ubiquitously expressed genes

For gene expression analysis, we used all the 67,651 gene models as the reference and mapped all raw reads from each library (454 Newbler GS Reference Mapper Version 2.6) with default parameters (Supplementary Table S7). We calculated the mean coverage to represent the expression level for each gene model and this method corrects biases in gene size and normalizes for the sequencing depth of each library (see Materials and Methods for details). Transcriptionally active cDNAs are identified as those having a mean coverage value greater than zero in at least one tissue or developmental stage. Using this criterion, we identified 56,112 (83 % of the total gene models) transcriptionally activated genes (Supplementary Table S8).

From these transcriptionally-active gene models, we identified 3,316 genes that are ubiquitously expressed in all tissues and at all developmental stages examined. When we ranked the shared gene models according to their expression levels, the top 100 genes from each tissue or developmental stage are pooled together to yield 382 genes as highly-expressed (Supplementary Table S9). Among them, we have ubiquitin and glyceraldehyde-3-phosphate dehydrogenase (GAPDH), the most frequently used genes as references for signal normalization in the literature (Ceol et al. 2004). This dataset is useful for transcriptional studies as controls or benchmarks. We understand that such an analysis may not allow thorough characterization of lowly-expressed transcripts so that the dataset most likely represents a lower bound of the ubiquitously expressed genes of date palm.

### Identification and comparative analysis of tissue-specific genes

**Fig. 6:**
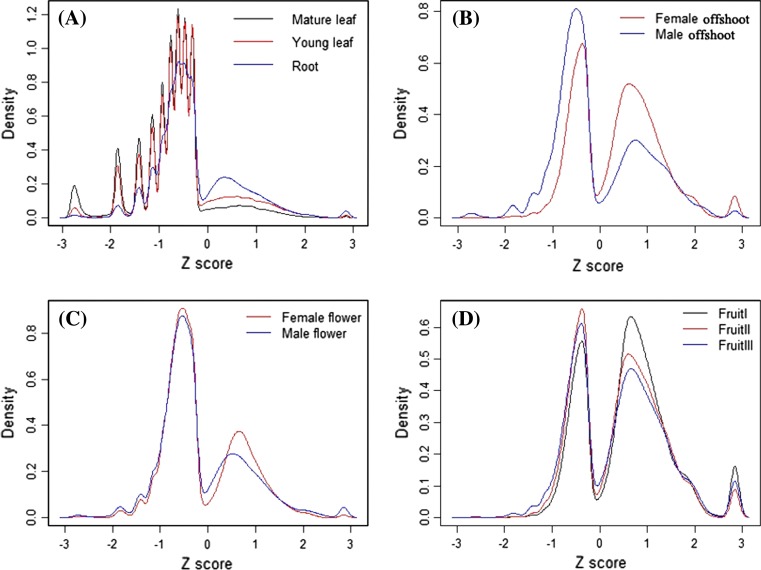
Relative gene expression levels (Z score) in different tissues. **a** Relative expression levels in mature leaf, young leaf, and root; **b** female and male offshoot; **c** female and male flowers, and **d** fruit at different developmental stages (I, II, and III)

Based on the expression value of each gene, we conducted a Z-score analysis to obtain insight into gene expression patterns (Severin et al. 2010; Schmid et al. 2005). The numerical value of Z-score stands for the standard deviation of expression levels of a given gene in a specific tissue based on the average expression level in all tissues. The Z-score analysis indicated that fruits are distinguished from other tissues by a three-peak expression pattern (Fig. 6). Transcriptions in the non-fruit tissues are less similar than those in the of fruit developmental stages, resulting in a smaller distribution of Z-scores and a small fraction of the gene models with Z-score values close to the positive extreme between 2.4 and 2.8 suggests a low specificity for the tissue. Setting 2.4 as a cut off value for the Z-score, we identified tissue-specific genes for each tissue or developmental stage. The number of tissue-specific genes in each tissue ranges from 58 in mature leaf to 2,099 in fruit-I. Further investigation of the tissue-specific genes revealed that most of them are uniquely-expressed in the corresponding tissues but are undetectable in others (Fig. 6). Furthermore, when analyzing the tissue-specific genes identified in each tissue based on their GO distribution (Supplementary Fig. S4), we found that similar tissues share a similar GO function distribution. In addition, many of these tissue-specific genes were annotated as functionally unknown; they are either newly evolved or fast-evolving paralogs. Comparing with other tissues, the fruit-I stage yielded the greatest number of tissue-specific genes, partly attributable to the high diversity of genes needed for fast cell division and morphological changes during this special phase of fruit development (Al-Farsi and Lee 2008; Al-Shahib and Marshall 2003). Overall, these tissue-specific genes provide important clues for future in-depth research on date palm flowering, fruiting, and development.

We estimated transcriptomic similarity between different tissues and developmental stages using Pearson correlation, taking all genes identified as transcriptionally activated into account. The resulted heatmap suggests three basic grouping of transcriptomes (Supplementary Fig. S5). The first group is comprised of leaf, offshoot, and root, and all of which are vegetative growing organs and rather limited in morphological development. The second group contains flowers only but both female and male flowers, which represents the development of reproductive organs and processes. Fruits formed a group by itself with an expression profile. In the fruit groups, fruit-II and fruit-III forms a clade together, which suggests that fruits in these two stages share a similar gene expression profile as compared to fruit-I. This clustering result is consistent with the developmental characteristics of fruits in a three-stage fashion. In the first stage, the fruit undergoes a rapid increase in size and weight (Al-Shahib and Marshall 2003); however, during the later stages (fruit-II and fruit-III), fruits mainly accumulate sugar and other components but have little increase in size and weight. Obviously, the expression profiles at genome level agree with the developmental relationship of the tissues. Similar results are also obtained in similar research of other plant species (Benedito et al. 2008; Wang et al. 2010).

### A detailed gene expression analysis between male and female flowers

**Fig. 7:**
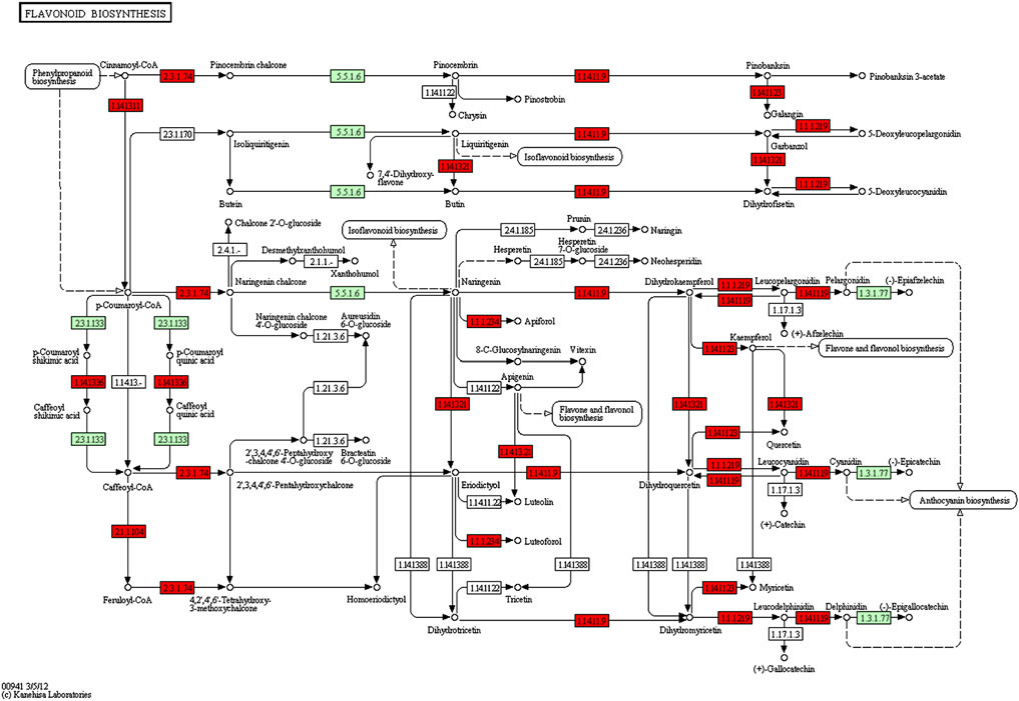
Regulatory changes in the pathway of flavonoid biosynthesis between female and male flowers. Colored rectangles correspond to genes involved in the pathway. Genes up-regulated in female flowers are highlighted in red over genes (in *jade*-*green*) detected in the pathway present in Arabidopsis but undetectable in our data due to lower sampling depth in our data

**Fig. 8:**
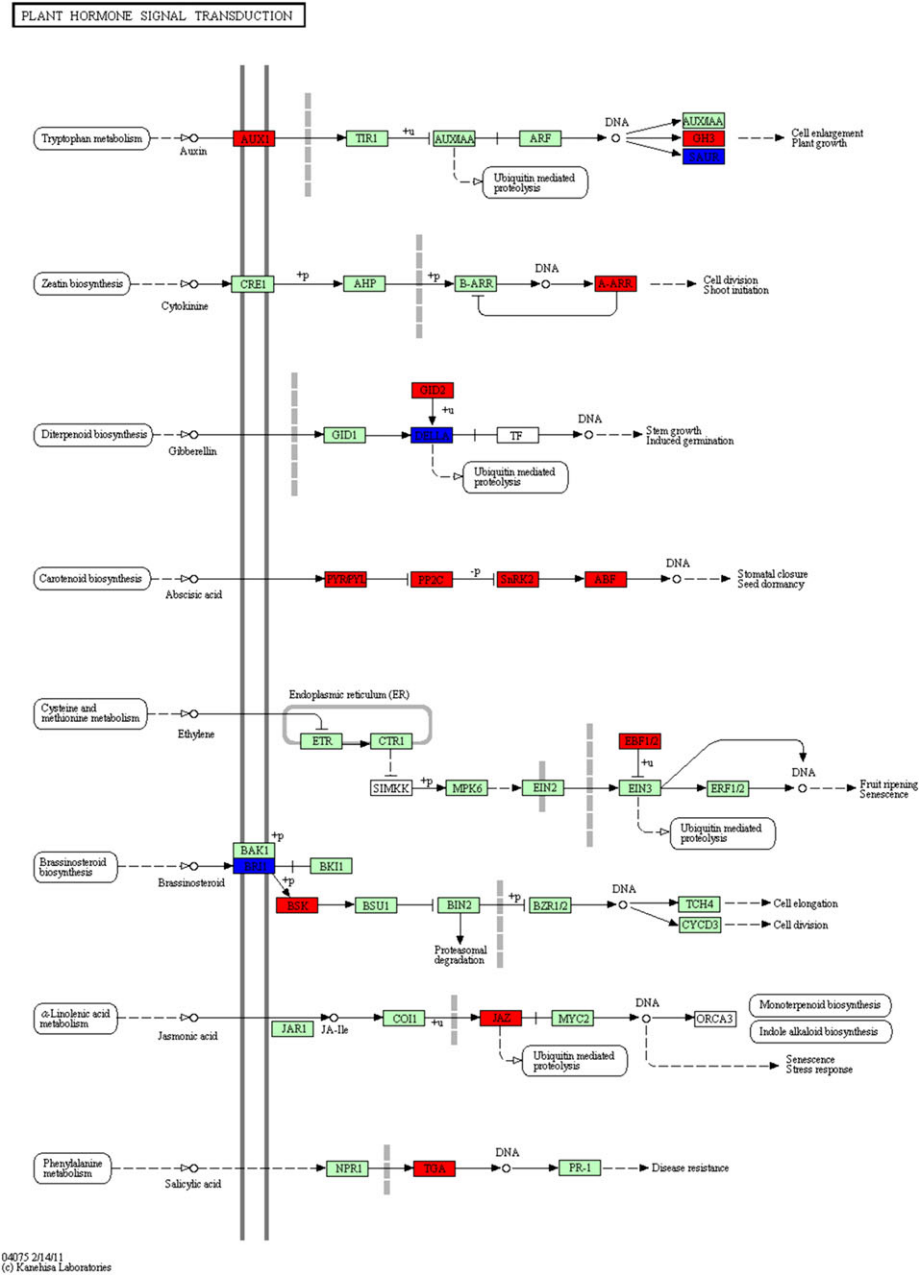
Regulatory changes in the pathway of plant hormone signal transduction between female and male flowers. Colored rectangles correspond to genes involved in the pathway. Up-regulated genes (*red*) in female flowers and those participated in the pathway present in Arabidopsis but undetected in our data (*jade*-*green*) are indicated

Aside from tissue specific analysis, we sought to explore gene expression profiles between male and female flowers. Based on expression values, using DEGseq (Wang et al. 2010), we identified differentially expressed genes (DEGs) between each group of tissues (*P* ≤ 0.001 as cut-off): 6,720 genes between male and female flowers. We defined 4,494 genes that are up-regulated in female flowers as compared to male flowers and 2,226 genes that are down-regulated in female flowers. Furthermore, we investigated the distribution of these DEGs according to KEGG pathways and found 1,038 genes associated with KO identifiers to have changed their expression level significantly between female and male flowers. Upon looking into all up-regulated DEG-related pathways in the female flower, we highlight here the flavonoid biosynthesis pathway (Fig. 7) since all key enzymes of this pathway were discovered in our data. Flavonoids are functionally related to the protection of UV irradiation (Schmelzer et al. 1988; van Tunen et al. 1988), and up-regulation is consistent with the biology of *P. dactylifera* that stands in the desert sun, enduring the strongest attacks from UV light. In addition, flavonoids play a crucial role in sexual reproduction in plants (Koes et al. 1990; van Tunen et al. 1990). The plant hormone signal transduction pathway is another one that changes significantly between female and male flowers (Fig. 8). Similar to the flavonoid biosynthesis pathway, most up-regulated DEGs in female flowers participated in this pathway. Transcription factor gene families are also interesting; there are many up-regulated DEGs in female flowers but not in male flowers, suggesting further developmental activities in the female flower such as fruiting. A highly-expressed TF gene, WRKY, is suspected to serve as a repressor in the male flower metabolic pathways.

We have also looked into the three early auxin-responsive genes that exhibit three distinct expression patterns in female and male flowers. The GH3 and SAUR genes are up-regulated in female and male flowers, respectively, whereas the expression level of AUX genes are up-regulated in female flowers. The phytohormone auxin exerts a pleiotropic effect on various aspects of plant growth and development, including cell elongation, cell division, differentiation, root initiation, apical dominance, and tropic responses. Auxin mediates these effects at the molecular level by altering the expression of numerous genes. The early auxin-responsive genes, which are specifically induced within minutes of auxin application, have been broadly grouped into three major classes: auxin/indoleacetic acid (Aux/IAA), GH3, and small auxin-up RNA (SAUR) gene families.

### Gene expression profiles of root, young and mature leaves

**Fig. 9:**
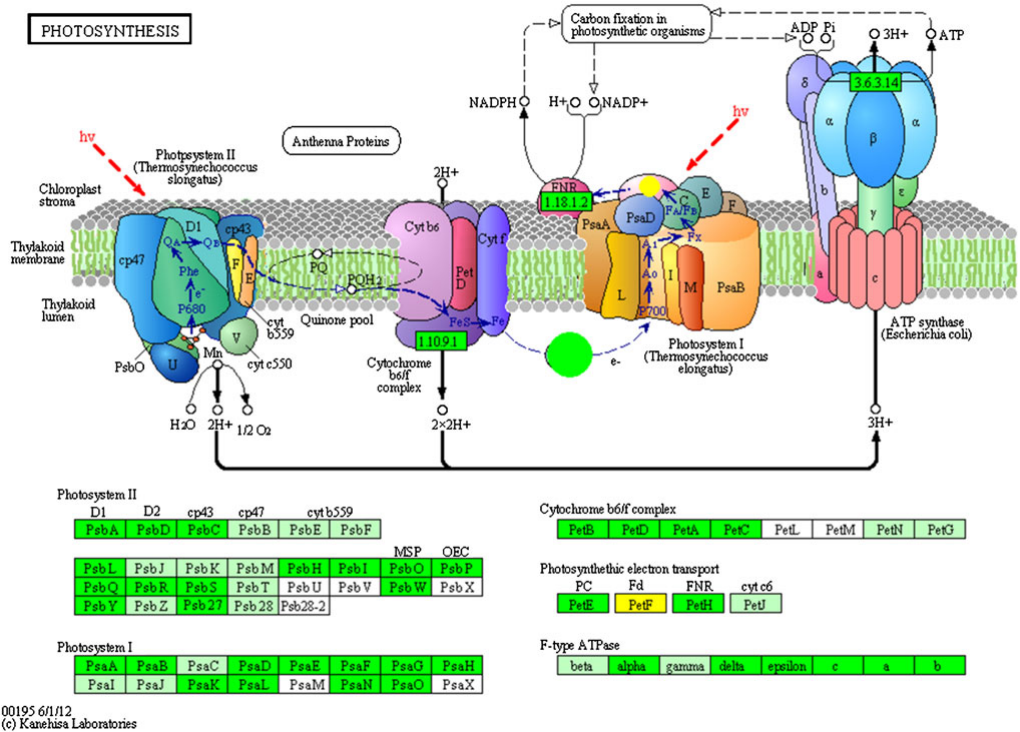
Up-regulated genes (*green*) in mature leaves present in the pathway of photosynthesis, and genes participated in the pathway present in Arabidopsis but undetected in our data (*jade*-*green*) are indicated

**Fig. 10:**
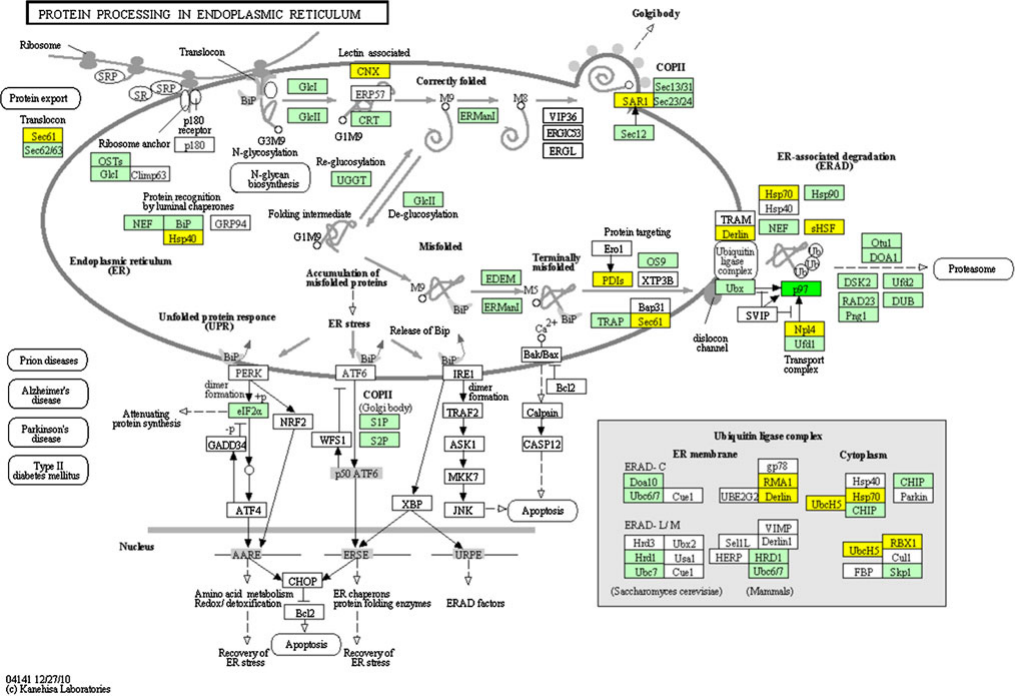
Up-regulated genes (*yellow*) in young leaves present in pathway of protein processing in endoplasmic reticulum, and genes participated in the pathway present in Arabidopsis but undetected in our data (*jade*-*gre*en) are indicated

In mature leaves, we identified 3,963 DEGs; 1,050 of them are up-regulated in mature leaves and 2,913 are down-regulated as compared to young leaves. A total of 473 genes associated with KO identifiers were found to change their expression level significantly between young and mature leaves. In the photosynthesis pathway (Fig. 9), all genes of the four multi-subunit membrane-protein complexes were found in our gene models: photosystem I, photosystem II, the cytochrome b6/f complex, and F-ATPase (Nelson and Yocum 2006), and as expected, in mature leaves the key metabolic pathway is photosynthesis. Another highlighted pathway is protein processing in endoplasmic reticulum. The endoplasmic reticulum is a subcellular organelle where proteins are folded with the help of luminal chaperones, and in this pathway, a series of genes were identified as up-regulated genes in young leaves (Fig. 10). The DEGs present in protein processing in endoplasmic reticulum are involved in earlier developmental stages of leaves and related to metabolic actions focusing on molecular synthesis instead of photosynthesis. According to the heatmap (Supplementary Fig. S5), young and mature leaves and root are clustered into one group, and we further investigated the DEGs between root and the leaves, the result showed that 3,589 are up-regulated in root as compared with leaves among 6,932 DEGs.

## Conclusions

Our study provides both *P. dactylifera* gene models in a large scale and expression profiles that cover a wide range of tissues and their developmental stages. This effort paves a way for future molecular and genetic studies of *P. dactylifera*, such as flower development, sex determination, and fruiting. The first glance of gene expression profiles allowed us to identify TF families that are specifically expressed at higher levels in male flower than in female flower. The dataset offers a valuable resource for future experimental work aiming at unraveling genetic regulatory networks that govern organ development and differentiation in *P. dactylifera*. Our future work includes generating a high-coverage transcriptomic dataset for all tissues and developmental stages and in-depth functional analysis and validation of genes in each *P. dactylifera* transcriptome.

## Materials and methods

### Plant materials

We collect eight tissues from male and female trees in different developmental stages of cultivar Khalas: root, offshoots from male and female trees, male and female flowers, young leaf (yellow), mature leaf (green), and fruit. The offshoots from male and female trees are the vegetative tissues arisen from the base of the mother palm. We collected the offshoots that are 13-month old and have a base diameter around 15 cm from healthy and pest-free mother palms. We subsequently dissected the offshoots to reveal the internal tissue. We collected fruit samples at seven developmental stages (Yin et al. 2012), constructed libraries, and acquired sequences to a high coverage. We subsequently combined the data into three basic groups for annotation and following-up analysis. We combined the data for the following three reasons. First, the young dates at F3 are also under-developed and shows an apple-green color; it is identical to kimri-II stage as described in the FAO description for date palm fruiting. Therefore, we clustered data from F3 with F1 and F2 as a new group fruitI. The larger dates in F4 are similar looking to those of F5 that are starch-accumulating and start to develop ripening color so that we pooled the sequences to become fruitII. At F6 and F7, the date colors become yellow and brown, respectively, but their sizes remain the same. The dates of both stages are actively accumulating monosaccharides so we merged the data into fruitIII. Second, our previous study identified a set of DEG genes which are characteristic of the fruiting stages and supports our new grouping scheme. Third, our current analysis is not to define DGEs for each tissues or developmental stages but to characterize gene models for future in-depth study of date palm gene expression. All samples were harvested from Al-Kharj, Saudi Arabia (24°08′54″N, 47°18′18″E). After thorough washing with distilled water, the samples were immediately frozen in liquid nitrogen and transported to the laboratory. The samples were stored at −80 °C until use.

### RNA extraction

Total RNA was extracted from 5-g tissue sample that was ground in liquid N_2_ according to a reported method (Bourgis et al. 2011). 20-ml preheated extraction buffer (65 °C) was quickly added to suspend the RNA and the mix was extracted twice with an equal volume of chloroform: isoamyl alcohol (24:1) and precipitated with 1/4 volume of 10 M LiCl and two volumes of ethanol overnight at 4 °C. The RNA was harvested by using centrifugation (13,000 rpm for 20 min) and dissolved in 500ul of SSTE buffer (10 mM Tris–HCl, pH 8.0, 1 mM EDTA, 1 M NaCl, 0.5 % (w/v) SDS). Another extract was performed with equal volume of chloroform: isoamyl alcohol (24:1) and the RNA was finally precipitated with two volumes of ethanol after treated with DNase I to digest DNA. The quality and purity of RNA were checked by using an Agilent 2100 Bioanalyzer (Agilent).

### Construction of near full-length cDNA library

Messenger RNA was extracted from total RNA based on a Qiagen Oligotex kit. We used at least 200-ng high-quality mRNA for each library construction. The RNA was fractioned by incubating at 70 °C in fragmentation buffer to yield a size range of 450 to 1,200 bp, and the resultant RNA was checked on an Agilent RNA 6000 Pico Chip (Agilent). We used a Rapid cDNA Library kits (Roche) for library construction. The library was heated at 95 °C for 2 min and chilled on ice immediately before emPCR. Sequencing was performed on a Roche/454 Genome Sequencer FLX Titanium Instrument by following its standard protocols.

### Sequence assembly and processing

After basic trimming for short or low quality reads, we pooled sequencing reads together for assembly, using 454 GS de novo Assembler (version 2.6) and parameters “-cdna -large -force -info -tr -ud -m -cpu -vt -vs”. Mitochondrial and chloroplast sequences from date palm were used as reference sequences to “-vt”.

Contamination removal and redundancy trimming involved five steps and all sequence output from the assembly was treated as input for the processing pipeline (Supplementary Fig. S2A). First, a perl script “seqclean” removes the sequences shorter than 150 bp. Second, contaminated sequences from microbes were identified by using BlastN against databases of microbial genomes downloaded from NCBI (ftp://ftp.ncbi.nih.gov/refseq/release/microbial/). The cutoff is set to *E* value ≤ 1 × 10^−10^, identity ≥80, and alignment length ≥90 %. Third, redundant contigs were trimmed by using a self-cross BlastN searching with a different cutoff (*E* value ≤ 1 × 10^−10^, identity ≥95 %, covered length ≥90 %). Fourth, compared with ribosomal RNA (rRNA) databases (http://www.girinst.org), any contigs and singletons meeting a specific cutoff (*E* value ≤ 1 × 10^−10^, identity ≥80 and covered length of query ≥80 %) were removed. Last, the remaining singletons were compared to unique contigs and those matching with contigs but smaller than their matched contigs were eliminated from further analysis.

### Analysis of non-redundant sequences

To select CDS from cDNA contigs, we first aligned all predicted CDS to the best Blast-hit proteins that were collected based on the blastX result of cDNA contigs versus UniProt proteins. We identify CDS according to following criteria: (1) homology with a known protein, (2) *E* value less than or equal to 1 × 10^−5^, (3) identity equal or larger than 30 %, (4) sequence length equal or larger than 30 amino acids, and (5) scoring as the highest among predicted CDS from all contigs. All putative transcription factor genes were examined through a BlastX search (*E* value ≤ 1 × 10^−5^) against Arabidopsis and rice transcription factor genes annotated in PlantTFDB (Zhang et al. 2011).

### Functional annotation

All date palm gene models were annotated according to the BlastX results against Plant UniProt and NR databases with an *E* value cutoff of 1 × 10^−5^. The unmatched gene models were further searched against Pfam database by using a local iprscan package. For a sequence with multiple Blast hits, we chose the one with the highest score as the best hit and annotated based on the gene. Gene ontology terms were retrieved for all protein identifiers from annotated sequences (ftp.geneontology.org/go/gene-associations/gene_association.goa_uniprot.gz). Furthermore, for all date palm sequences we used the KEGG Automatic Annotation Server (http://www.genome.jp/kaas-bin/kaas_main?modeest_b) to identify KEGG orthologs with default parameters. We compared our gene models with annotated genes from two monocot plants (*O. sativa* japonica and *S. bicolor*) and two dicot plants (*A. thaliana* and *V. vinifera*) using BlastX (1 × 10^−10^). We also compared our data to those released by Weill Cornell Medical College in Qatar (http://qatar-weill.cornell.edu/research/datepalmGenome/index.html).

### Gene expression analysis

We first mapped raw reads from each tissue (or stage) back to our gene models using 454 Newbler GS Reference Mapper (Version 2.6) with default parameters. Second, we calculated the mean coverage of a single gene models in each tissue (or stage) following the formula: ${\text{C}}_{\text{m}} = \frac{{10^{9} \times {\text{n}}}}{{{\text{N }} \times {\text{L}}}}$, where ${\text{C}}_{\text{m}}$ is the mean coverage of the gene models, n is the mapped bases of the gene models in a single library, N is the total mapped bases of the corresponding library, and L is the length of the gene models. This method corrects biases in gene size and normalizes for sequencing depth of each library. Third, setting ${\text{C}}_{\text{m}}$>0 as the cut-off value, we identified transcriptionally activated genes in each tissue or stage and obtained gene expression profiles for each tissue or developmental stage.

### Tissue-specificity analysis for each tissue

Based on gene expression profile for each tissue, we quantitated transcriptional activities for each gene and each tissue. We set 0.001 as the cut-off *P* value and Benjamini correction as the control of false discovery rate. To identify tissue-specific genes, we calculated Z score for each gene in every single tissue based on expression value calculated above ${\text{C}}_{\text{m}}$ (value). The C_m_ value was log_2_-transformed and a Z score value was calculated according to $Z = \frac{x - \mu }{\sigma }$. In the formula$x$ , is the log_2_-transformed C_m_ value of a gene in a special tissue, $\mu$ is the mean of log_2_-transformed C_m_ values in all tissues, and $\sigma$ is the standard deviation of log_2_ –transformed C_m_ values in all tissues.

## Electronic supplementary material

Below is the link to the electronic supplementary material.

Supplementary material 1 (DOC 81 kb)

Supplementary material 2 (DOCX 14 kb)

Supplementary material 3 (DOCX 15 kb)

Supplementary material 4 (DOCX 15 kb)

Supplementary material 5 (DOCX 16 kb)

Supplementary material 6 (XLSX 59 kb)

Supplementary material 7 (DOCX 15 kb)

Supplementary material 8 (XLSX 5097 kb)

Supplementary material 9 (XLSX 41 kb)

Supplementary material 10 (XLSX 395 kb)

Supplementary material 11 (XLSX 575 kb)

Supplementary material 12 (DOCX 17 kb)

Supplementary material 13 (XLSX 3982 kb)

Supplementary material 14 (TIFF 121 kb)

Supplementary material 15 (TIFF 349 kb)

Supplementary material 16 (TIFF 116 kb)

Supplementary material 17 (TIFF 464 kb)

Supplementary material 18 (TIFF 209 kb)
