# Supplementary material for: Large-scale collection and annotation of gene models for date palm (Phoenix dactylifera, L.)
Source: Plant Mol Biol. 2012 Jun 27;79(6):521–36. doi: 10.1007/s11103-012-9924-z (PMC3402680; doi:10.1007/s11103-012-9924-z)
Supplement: Supplementary file 1 — Supplementary material 1 (DOC 81 kb) [file 11103_2012_9924_MOESM1_ESM.doc]

Codon usage estimated from date palm, rice, and Arabidopsis FL-cDNAs.

| Codon | Amino Acid | Fraction* | | |
| --- | --- | --- | --- | --- |
|  |  | Date palm | Rice | Arabidopsis |
| GCA | A | 0.35 | 0.19 | 0.25 |
| GCC | A | 0.25 | 0.33 | 0.17 |
| GCG | A | 0.16 | 0.27 | 0.14 |
| GCT | A | 0.22 | 0.22 | 0.44 |
| TGC | C | 0.59 | 0.69 | 0.20 |
| TGT | C | 0.40 | 0.32 | 0.58 |
| GAC | D | 0.42 | 0.54 | 0.33 |
| GAT | D | 0.57 | 0.46 | 0.67 |
| GAA | E | 0.50 | 0.34 | 0.50 |
| GAG | E | 0.49 | 0.66 | 0.50 |
| TTC | F | 0.57 | 0.65 | 0.51 |
| TTT | F | 0.42 | 0.35 | 0.49 |
| GGA | G | 0.32 | 0.19 | 0.36 |
| GGC | G | 0.23 | 0.40 | 0.15 |
| GGG | G | 0.26 | 0.21 | 0.15 |
| GGT | G | 0.17 | 0.20 | 0.34 |
| CAC | H | 0.44 | 0.56 | 0.41 |
| CAT | H | 0.55 | 0.44 | 0.59 |
| ATA | I | 0.31 | 0.19 | 0.23 |
| ATC | I | 0.37 | 0.48 | 0.37 |
| ATT | I | 0.30 | 0.33 | 0.40 |
| AAA | K | 0.45 | 0.30 | 0.48 |
| AAG | K | 0.54 | 0.70 | 0.52 |
| CTA | L | 0.10 | 0.07 | 0.10 |
| CTC | L | 0.15 | 0.30 | 0.19 |
| CTG | L | 0.20 | 0.25 | 0.11 |
| CTT | L | 0.13 | 0.17 | 0.26 |
| TTA | L | 0.13 | 0.06 | 0.13 |
| TTG | L | 0.27 | 0.15 | 0.22 |
| ATG | M | 1.00 | 1.00 | 1.00 |
| AAC | N | 0.48 | 0.57 | 0.50 |
| AAT | N | 0.51 | 0.43 | 0.50 |
| CCA | P | 0.37 | 0.25 | 0.32 |
| CCC | P | 0.20 | 0.22 | 0.12 |
| CCG | P | 0.20 | 0.30 | 0.19 |
| CCT | P | 0.21 | 0.24 | 0.38 |
| CAA | Q | 0.49 | 0.35 | 0.55 |
| CAG | Q | 0.50 | 0.65 | 0.46 |
| AGA | R | 0.31 | 0.15 | 0.34 |
| AGG | R | 0.29 | 0.24 | 0.19 |
| CGA | R | 0.10 | 0.08 | 0.12 |
| CGC | R | 0.08 | 0.26 | 0.08 |
| CGG | R | 0.14 | 0.17 | 0.09 |
| CGT | R | 0.05 | 0.10 | 0.17 |
| AGC | S | 0.15 | 0.20 | 0.13 |
| AGT | S | 0.09 | 0.11 | 0.14 |
| TCA | S | 0.24 | 0.16 | 0.20 |
| TCC | S | 0.20 | 0.21 | 0.14 |
| TCG | S | 0.12 | 0.16 | 0.11 |
| TCT | S | 0.17 | 0.16 | 0.29 |
| ACA | T | 0.36 | 0.24 | 0.29 |
| ACC | T | 0.26 | 0.32 | 0.21 |
| ACG | T | 0.15 | 0.23 | 0.16 |
| ACT | T | 0.21 | 0.22 | 0.34 |
| GTA | V | 0.19 | 0.10 | 0.14 |
| GTC | V | 0.21 | 0.31 | 0.20 |
| GTG | V | 0.36 | 0.36 | 0.25 |
| GTT | V | 0.22 | 0.23 | 0.41 |
| TGG | W | 1.00 | 1.00 | 1.00 |
| TAC | Y | 0.45 | 0.63 | 0.50 |
| TAT | Y | 0.54 | 0.37 | 0.50 |
| TAA | stop | 0.23 | 0.25 | 0.36 |
| TGA | stop | 0.24 | 0.46 | 0.44 |
| TAG | stop | 0.51 | 0.27 | 0.20 |
