# Supplementary material for: Large-scale collection and annotation of gene models for date palm (Phoenix dactylifera, L.)
Source: Plant Mol Biol. 2012 Jun 27;79(6):521–36. doi: 10.1007/s11103-012-9924-z (PMC3402680; doi:10.1007/s11103-012-9924-z)
Supplement: Supplementary file 2 — Supplementary material 2 (DOCX 14 kb) [file 11103_2012_9924_MOESM2_ESM.docx]

**SSR analysis of 30,854 gene models**

|  | Di-NR | Tri-NR | Tetra-NR | Penta-NR | Hexa-NR | Total | % |
| --- | --- | --- | --- | --- | --- | --- | --- |
| 5’UTR | 502 | 189 | 59 | 18 | 4 | 772 | 37.4 |
| CDS | 13 | 483 | 4 | 5 | 18 | 523 | 25.3 |
| 3’UTR | 471 | 197 | 73 | 16 | 10 | 767 | 37.1 |
| TOTAL | 986 | 869 | 136 | 39 | 32 | 2,062 |  |
| % | 47.8 | 42.1 | 6.5 | 1.8 | 1.5 |  |  |
