# Supplementary material for: Large-scale collection and annotation of gene models for date palm (Phoenix dactylifera, L.)
Source: Plant Mol Biol. 2012 Jun 27;79(6):521–36. doi: 10.1007/s11103-012-9924-z (PMC3402680; doi:10.1007/s11103-012-9924-z)
Supplement: Supplementary file 3 — Supplementary material 3 (DOCX 15 kb) [file 11103_2012_9924_MOESM3_ESM.docx]

**TE analysis of 30,854 gene models^1^**

| Type 1: DNA transposons Type 2: Retrotransposons | | | | | | | | |
| --- | --- | --- | --- | --- | --- | --- | --- | --- |
| DNA transposons | | | Non-LTR retrotransposons | | | LTR retrotransposons | | |
| TE | Count |  | TE | Count |  | TE | Count | |
| En/Spm | 779 |  | RTE | 21 |  | *Copia* | 604 | |
| hAT | 133 |  | NeSL | 4 |  | *Gypsy* | 714 | |
| Harbinger | 71 |  | L1 | 195 |  | LTR | 21 | |
| Helitron | 138 |  | Non-LTR | 1 |  |  |  | |
| Mariner/Tc1 | 1 |  |  |  |  |  |  | |
| MuDR | 190 |  |  |  |  |  |  | |
| DNA | 1,306 |  |  |  |  |  |  |  |
| Total | 2,618 |  |  | 221 |  |  | 1,339 | |

Note: 1. The number of gene models choozen for the analysis is a non-redundant annotated set.
