# Supplementary material for: Large-scale collection and annotation of gene models for date palm (Phoenix dactylifera, L.)
Source: Plant Mol Biol. 2012 Jun 27;79(6):521–36. doi: 10.1007/s11103-012-9924-z (PMC3402680; doi:10.1007/s11103-012-9924-z)
Supplement: Supplementary file 4 — Supplementary material 4 (DOCX 15 kb) [file 11103_2012_9924_MOESM4_ESM.docx]

GO term distribution of *P. dactylifera* gene models

| **Biological process** | % |
| --- | --- |
| Primary metabolic process | 41.2 |
| Cellular metabolic process | 39.9 |
| Macromolecule metabolic process | 30.3 |
| Biosynthetic process | 18.0 |
| Nitrogen compound metabolic process | 17.7 |
| Establishment of localization | 9.7 |
| Transport | 9.7 |
| Regulation of biological process | 7.9 |
| Regulation of cellular process | 7.7 |
| Oxidation reduction | 6.7 |
| **Molecular function** | % |
| Nucleic acid binding | 22.4 |
| Nucleotide binding | 21.7 |
| Ion binding | 19.7 |
| Transferase activity | 18.1 |
| Hydrolase activity | 16.7 |
| Nucleoside binding | 15.5 |
| Oxidoreductase activity | 7.7 |
| Protein binding | 4.3 |
| Substrate-specific transporter activity | 3.9 |
| Transmembrane transporter activity | 3.7 |
| **Cellular component** | % |
| Cell part | 39.6 |
| Intracellular | 27.8 |
| Intracellular part | 24.2 |
| Intracellular organelle | 19.0 |
| Membrane-bounded organelle | 14.9 |
| Membrane | 14.4 |
| Membrane part | 10.0 |
| Organelle part | 5.5 |
| Intracellular organelle part | 5.5 |
| Non-membrane-bounded organelle | 5.3 |
