# Supplementary material for: Large-scale collection and annotation of gene models for date palm (Phoenix dactylifera, L.)
Source: Plant Mol Biol. 2012 Jun 27;79(6):521–36. doi: 10.1007/s11103-012-9924-z (PMC3402680; doi:10.1007/s11103-012-9924-z)
Supplement: Supplementary file 5 — Supplementary material 5 (DOCX 16 kb) [file 11103_2012_9924_MOESM5_ESM.docx]

Top 10 representative transcription factors (TFs) in date palm, Arabidopsis, and rice.

| Date palm | | | Arabidopsis | | | Japonica rice | | |
| --- | --- | --- | --- | --- | --- | --- | --- | --- |
| TF | Count | % | TF | Count | % | TF | Count | % |
| bHLH | 128 | 9.36 | bHLH | 194 | 9.59 | bHLH | 225 | 9.23 |
| MYB | 85 | 6.21 | MYB | 159 | 7.86 | MYB | 186 | 7.63 |
| NAC | 65 | 4.75 | NAC | 135 | 6.67 | NAC | 186 | 7.63 |
| ERF | 78 | 5.70 | ERF | 132 | 6.52 | ERF | 163 | 6.69 |
| C2H2 | 82 | 5.99 | C2H2 | 104 | 5.14 | C2H2 | 110 | 4.51 |
| bZIP | 70 | 5.12 | bZIP | 101 | 4.99 | bZIP | 140 | 5.74 |
| WRKY | 56 | 4.09 | WRKY | 89 | 4.40 | WRKY | 137 | 5.62 |
| MYB_related | 55 | 4.02 | MYB_related | 85 | 4.20 | MYB_related | 108 | 4.43 |
| C3H | 72 | 5.26 | M-type | 73 | 3.61 | C3H | 82 | 3.36 |
| G2-like | 44 | 3.22 | B3 | 71 | 3.51 | GRAS | 74 | 3.04 |
