# Supplementary material for: Large-scale collection and annotation of gene models for date palm (Phoenix dactylifera, L.)
Source: Plant Mol Biol. 2012 Jun 27;79(6):521–36. doi: 10.1007/s11103-012-9924-z (PMC3402680; doi:10.1007/s11103-012-9924-z)
Supplement: Supplementary file 7 — Supplementary material 7 (DOCX 15 kb) [file 11103_2012_9924_MOESM7_ESM.docx]

Reference mapping results. All raw reads in each tissue or developmental stage were mapped onto 67,651 contigs.

|  | Fruit-I | Fruit-II | Fruit-III | Male flower | Female flower | Male offshoot | Female offshoot | Young leaf  （Yellow） | Mature leaf  （Green） | Root |
| --- | --- | --- | --- | --- | --- | --- | --- | --- | --- | --- |
| Total reads | 3,142,488 | 2,230,582 | 2,450,576 | 775,727 | 571,170 | 324,461 | 2,451,459 | 697,044 | 1,060,675 | 1,008,818 |
| Total bp | 1,130,324,003 | 811,038,041 | 889,091,864 | 222,993,399 | 181,255,968 | 120,785,676 | 822,605,343 | 256,961,919 | 372,323,537 | 358,654,438 |
| Unique mapping reads | 1,326,499 | 833,983 | 948,539 | 391,855 | 253,193 | 119,434 | 1,025,317 | 78,045 | 74,099 | 112,611 |
| Multiple mapping reads | 1,131,769 | 801,478 | 840,796 | 253,322 | 194,565 | 87,184 | 954,810 | 223,672 | 245,840 | 301,540 |
| Unique mapping bp | 576,768,680 | 381,720,951 | 433,350,166 | 131,097,783 | 97,108,962 | 57,024,429 | 430,152,956 | 66,121,839 | 58,157,190 | 96,552,926 |
| Expressed contig number | 40,745 | 37,359 | 36,703 | 22,221 | 21,579 | 23,455 | 36,758 | 13,394 | 8,531 | 18,904 |
