# Supplementary material for: Large-scale collection and annotation of gene models for date palm (Phoenix dactylifera, L.)
Source: Plant Mol Biol. 2012 Jun 27;79(6):521–36. doi: 10.1007/s11103-012-9924-z (PMC3402680; doi:10.1007/s11103-012-9924-z)
Supplement: Supplementary file 12 — Supplementary material 12 (DOCX 17 kb) [file 11103_2012_9924_MOESM12_ESM.docx]

**Summary of gene models in different related datasets.**

| Name | Gene/contigs Number | Matched Number  (Object) | Matched Number  (Query) | Unmatched  (Query) |
| --- | --- | --- | --- | --- |
|  |  |  |  |  |
| Genes date palm (predicted, Qatar) | 28,889 | 23,810 | 49,622 | 18,029 |
| Fruit of date palm (cDNAs, Tunis) | 37,048 | 20,701 | 50,834 | 16,817 |
| Fruit of oil palm (cDNAs, Tunis) | 33,841 | 18,657 | 48,229 | 19,322 |
| Leaf of oil palm (cDNAs, Tunis) | 7,854 | 6,404 | 26,410 | 41,241 |
