# Supplementary figures and images for: Large-scale collection and annotation of gene models for date palm (Phoenix dactylifera, L.)
Source: Plant Mol Biol. 2012 Jun 27;79(6):521–36. doi: 10.1007/s11103-012-9924-z (PMC3402680; doi:10.1007/s11103-012-9924-z)

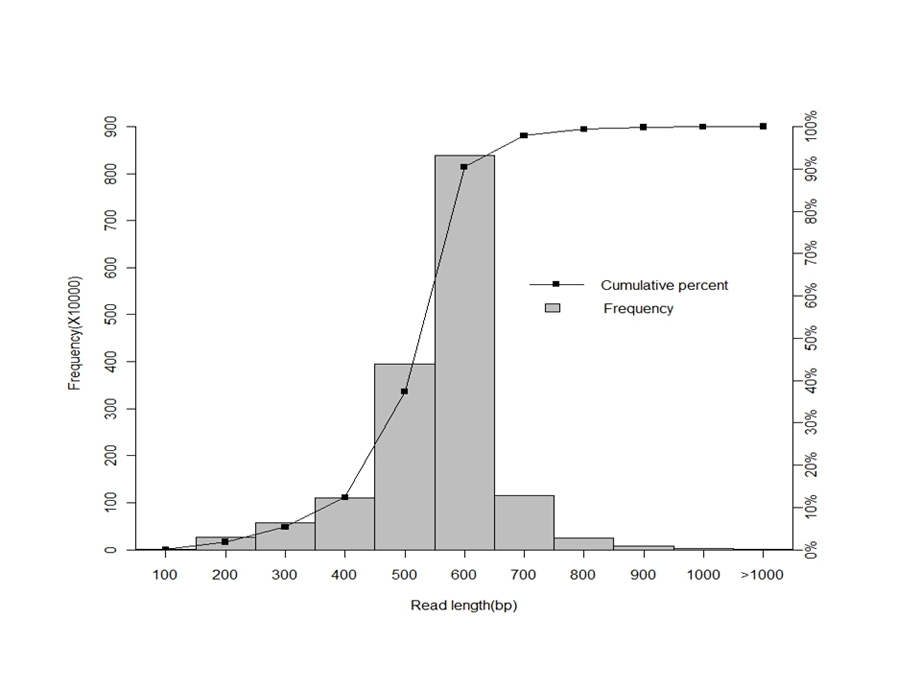

Supplement: Supplementary file 14 — Supplementary material 14 (TIFF 121 kb) [file 11103_2012_9924_MOESM14_ESM.tif]

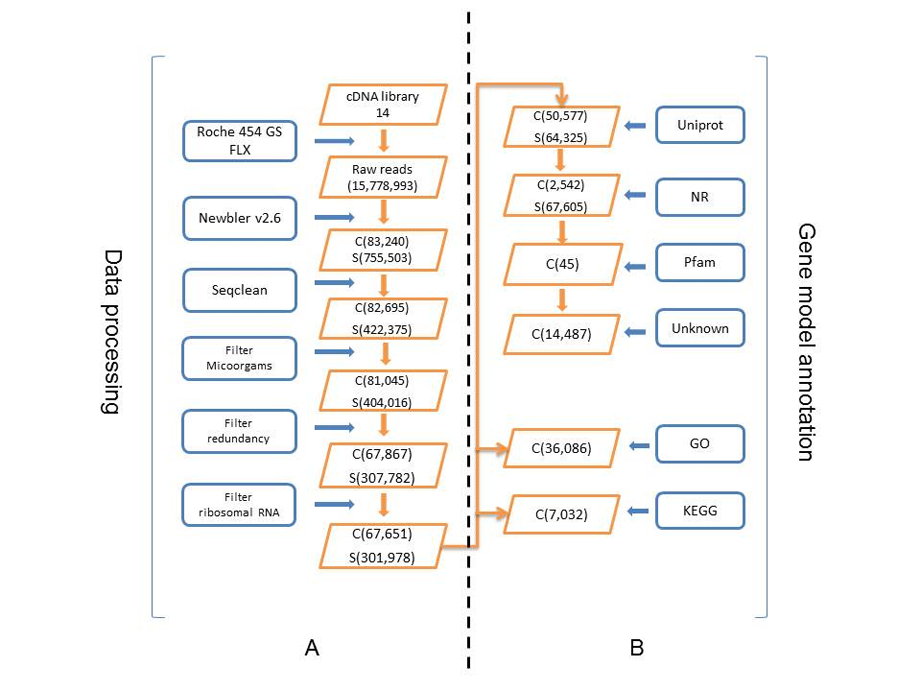

Supplement: Supplementary file 15 — Supplementary material 15 (TIFF 349 kb) [file 11103_2012_9924_MOESM15_ESM.tif]

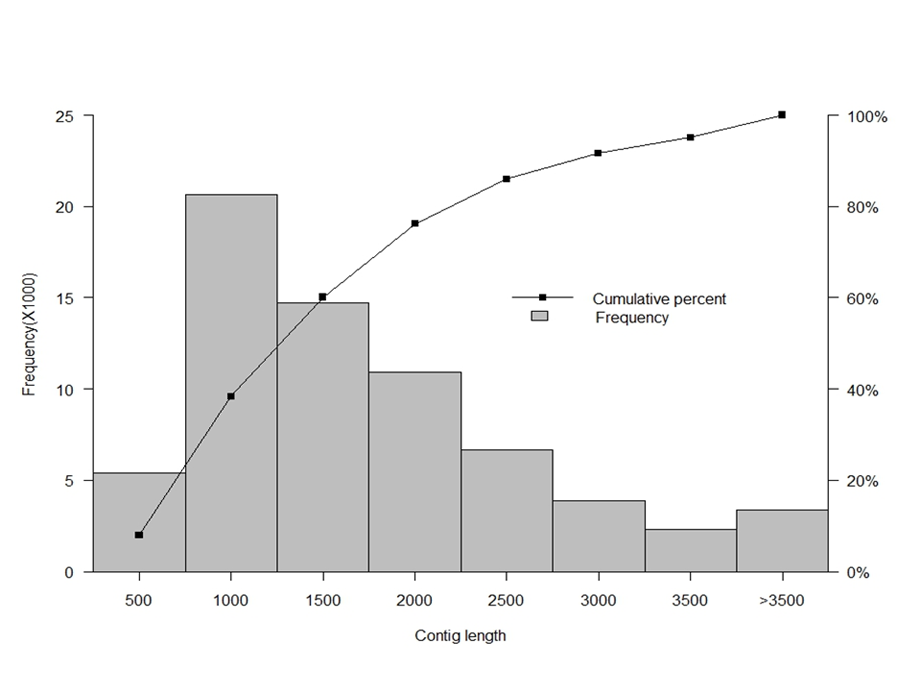

Supplement: Supplementary file 16 — Supplementary material 16 (TIFF 116 kb) [file 11103_2012_9924_MOESM16_ESM.tif]

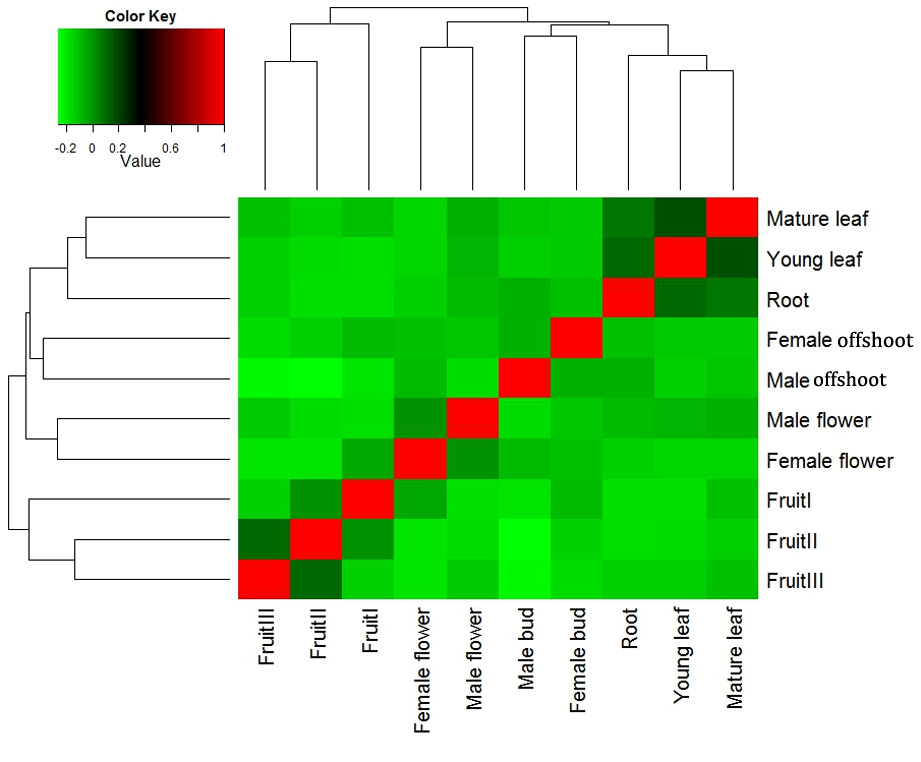

Supplement: Supplementary file 18 — Supplementary material 18 (TIFF 209 kb) [file 11103_2012_9924_MOESM18_ESM.tif]
